# Systematic review for the development of a pharmaceutical and medical products prioritization framework

**DOI:** 10.1186/s40545-019-0181-2

**Published:** 2019-08-21

**Authors:** Alberto Frutos Pérez-Surio, Mercedes Gimeno-Gracia, Ma. Aránzazu Alcácera López, Ma. Asunción Sagredo Samanes, Ma. del Puerto Pardo Jario, Ma. del Tránsito Salvador Gómez

**Affiliations:** 1 Department of Hospital Pharmacy, University Clinical Hospital Lozano Blesa. Avda. San Juan Bosco 15, 50009 Zaragoza, Spain; 2 Department of Microbiology, Preventive Medicine and Public Health, University of Zaragoza, C/Domingo Miral s/n 50009, Zaragoza, Spain; 3 IIS Aragón, Zaragoza, Spain

**Keywords:** Pharmaceuticals, Medical products, Health technology assessment, Decision-making, Health priorities, Criteria resource allocation

## Abstract

**Objective:**

To identify and analyze the criteria, approaches, and conceptual frameworks, used for national/international priority setting.

**Data sources:**

We performed a search of the main biomedical databases (Medline/PubMed, Embase, Centre for Reviews and Dissemination, and Cochrane), and we reviewed assessment agency websites, among other sources.

**Study design:**

An systematic review of the literature was carried out.

**Data collection:**

Eligibility criteria for inclusion were based on set of predefined criteria. Systematic reviews and/or qualitative studies (interviews, surveys, expert consensus, etc) that aimed to identify prioritization criteria or develop general operational frameworks for the selection of health priorities were included. A critical analysis is made of all the aspects that may be useful for any public body that intends to establish priorities in health.

**Principal findings:**

We found that there are no standardized criteria for priority setting, although common trends have been identified regarding key elements. Eight key domains were identified: 1) need for intervention; 2) health outcomes; 3) type of benefit of the intervention; 4) economic consequences; 5) existing knowledge on the intervention/quality and uncertainties of the regarding evidence; 6) implementation and complexity of the intervention/feasibility; 7) justice and ethics; and 8) overall context.

**Conclusions:**

Our review provides a thorough analysis of the relevant issues and offers key recommendations regarding considerations for developing a national prioritization framework. Findings are envisioned to be useful for different public organizations that are aiming to establish healthcare priorities.

**Electronic supplementary material:**

The online version of this article (10.1186/s40545-019-0181-2) contains supplementary material, which is available to authorized users.

## Introduction

A health technology is defined as an intervention that may be used to promote health, to prevent, diagnose or treat acute or chronic disease, or for rehabilitation. Health technologies include pharmaceuticals, devices, procedures and organizational systems used in health care [1].

The World Health Organization (WHO) state that defines health technology assessment (HTA) refers to the systematic evaluation of properties, effects, and/or impacts of health technology [2]. It is a multidisciplinary process to evaluate the social, economic, organizational and ethical issues of a health intervention or health technology [3].

The main purpose of conducting our assessment is to develop an explicit priority setting methodology to support decision-making regarding Medicines and Medical Devices to be included in Hospital Pharmacy practice. The development of a comprehensive prioritization system is the outcome essential for an important benefit to the healthcare system [4]. The aim of this research is to identify and analyze the processes and decision criteria used internationally for priority setting in order to establish a comprehensive set of strategic criteria for starting point for the development of a Medicines and Medical Devices prioritization framework.

## Methods

A systematic search of the literature was carried out in December 2017, in the main biomedical electronic databases: Medline/PubMed, Embase, Centre for Reviews and Dissemination (CRD), and Cochrane. For this, a specific search strategy was designed combining the terms: “medicine”, “technology assessment, biomedical”, “technology”, “intervention” with “priority”, “prioriti*”, “selection” with “criteria”, “Setting”, “approach*”and “procedure*”. The detailed search terms for different electronic databases is listed in Additional file 1. Eligibility criteria for inclusion / exclusion were based on set of predefined criteria (Table 1). Systematic reviews and/or qualitative studies (interviews, surveys, expert consensus, etc) that aimed to identify prioritization criteria or develop general operational frameworks for the selection of health priorities were included. The web pages of the international agencies belonging to EUnetHTA and INAHTA were reviewed, and manually searched in the main scientific journal of the specialty (*International Journal of Technology Assessment Health Care*) [5–8]. In addition, a general search was carried out in the Google and Google Scholar search engine to locate gray literature, and bibliographic citations of included studies were reviewed. For perusal of the complete text, we selected records in which any type of pharmaceutical or medical product was assessed. It was not considered relevant to apply a methodological quality scale or gradation of evidence when not addressing a clinical research question. Data of the studies were analyzed and synthesized qualitatively.

**Table 1:** Criteria for the selection of studies

Types of publications	Inclusion: articles published in peer-reviewed journals and documents published on official websites Exclusion: communications to congresses, letters to the editor, editorials, commentaries
Types of articles / documents	**Inclusion:** original articles (qualitative studies, surveys, interviews, consensus methods, panels of experts), systematic reviews, formulations of conceptual frameworks based on evidence / expert opinion, guidelines / procedures manuals or dissemination articles **Exclusion:** opinion articles
Scope	**Inclusion:** articles that address the identification, selection or categorization of prioritization criteria, define or propose strategic or operational frameworks for the selection of health priorities, or describe the mechanisms or processes employed by different national and international agencies to prioritize medicinal products and medical devices **Exclusion:** methodological developments
Area	**Inclusion:** prioritization processes designed to inform reimbursement and financing policies **Exclusion:** prioritization processes aimed at other areas (inform the development of guidelines, clinical protocols, detection systems of new or emerging technologies, observation of technologies, disinvestment, health technology assessment units, etc.)
Language	**Inclusion:** English. Also, Spanish, Italian, French and Portuguese.
Time frame	Unlimited

## Results

**Fig. 1:**
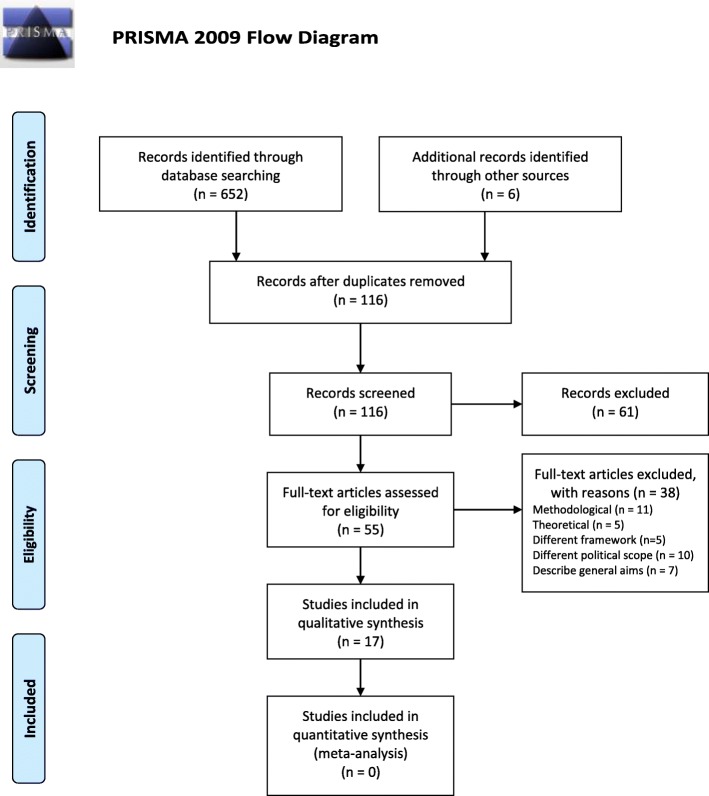
PRISMA 2009 Flow Diagram

A total of 17 documents complied with eligibility criteria, out of which 15 were published in scientific journals [9–23] and two elsewere [24, 25]. Fig. 1 details the selection process of the articles and the reasons for exclusion of potentially relevant articles. The studies showed great heterogeneity. A total of 56 potentially relevant priority setting criteria were identified, which could be grouped in eight categories: 1) Need for intervention; 2) Outcomes of intervention; 3) Type of benefit; 4) Economic consequences; 5) Existing knowledge/quality of evidence and uncertainties; 6) Implementation complexity/feasibility; 7) Justice and equity; and 8) Context. Table 2 describes these eight categories. Table 2 describes the domains and general criteria identified or proposed in these studies, detailing the conceptual terms used to classify them.

**Table 2:** Priority setting criteria. Main sources

Domain	Considered criteria	Alternative categorizations / subgroupings
Need for intervention	-Severity of the disease / condition	**Target disease**
	- Population size	- Severity of the disease
	-Unmet need / availability of alternatives	-Determinants of the disease
		-Burden of illness / threat to life
		-Economic burden of the disease
		-Epidemiology
		**Therapeutic context**
		-Therapeutic alternatives / need not met
		-Need
		-Clinical practice guidelines and protocols
		-Existing use
Health results	-Benefits in health / clinical	**Clinical benefits**
	-Efficacy / Effectiveness	- General clinical benefits
	- Safety / tolerability	-Effect on mortality
	-Health perceived by the patient	-Effect on longevity
	-Quality of care	-Effect on quality of life
		**Health perceived by the patient:**
		-Quality of life
		- Autonomy
		- Impact on dignity
		- Improved use / administration
		**Adequacy**
		- Efficiency and safety
		- Effectiveness
		**Response level**
		- Quality of care received by the patient
		- Burden of disease
Type of benefit of the intervention	-Preventive benefits	
	-Therapeutic benefits	
Economic consequences / economic impact	-Costs of the intervention	**Efficiency**
	- Medical / health costs	- Cost effectiveness / benefit
	- Non-medical costs (productivity, cost, patients, caregivers)	- Budget impact
	-Impoverishment for the patient	- Costs
	- Budget impact	**Financing**
	-Financial impact	- Unit cost
	-Impact on productivity	- Budget impact
	-Impact on other services	- Financing agent
	-Efficiency and opportunity cost	**Cost of opportunity and affordability (context-dependent criteria)**
	-Cost-effectiveness	- Opportunity cost and if the system can afford it
Existing knowledge about the intervention / Quality and uncertainty of the evidence	-Evidence available	**Other considerations**
	-Quality of the evidence	Quality of clinical and economic evidence
	-Relevance of the evidence	Consistency with strategic aspects
	-Uncertainty of the evidence	
	-Expert consensus / clinical practice guidelines	
Implementation and complexity of the intervention / Feasibility	-Regulatory requirements / legislation	
	-Organizational requirements	
	-Technological requirements	
	-Requirements of personnel	
	-Training / personal skills requirements	
	-Information requirements	
	-Implementation flexibility	
	-Features of the intervention	
	-Appropriate use	
	-Barriers and acceptability	
	-Integration and efficiency of the system	
	-Sustainability	
	-Accessibility to the population	
Ethics and justice	-Population priorities	**Priority, ethics and justice**
	-Access	- Low socioeconomic status
	-Vulnerability	- Children (0–5 years old or elderly)
	-Utility	- Subjects of productive age
	-Solidarity	- Women in productive age
	-Ethics and moral aspects	- Remote communities
		- Therapeutic specific areas
		- Response behavior
		- Rare diseases
		- Specific groups of patients
		**Equity**
		- General
		- Accessibility
		- Accessibility for the individual
		**Other ethical and social values**
		- Autonomy
		- Value public health
		- Impact in future generations
		- Risk social and financial
		- Catastrophic sanitary cost
		- Economic productivity and care for third parties
		- Rare diseases-Population priorities
Global context	-Mandate and mission of the health system	**Governance / leadership**
	-Alignment with regulations and strategies	- Congruence with prior prioritization
	- Global priorities / alignment with priority lines (vulnerable groups, disabled, diseases, rare, etc.)	- Cultural acceptability
	-Financial Restriction	- Political acceptability
	-Incentives	- Acceptability of interest groups
	-Political aspects	- Legal Barriers
	- Historical aspects	
	-Cultural aspects	
	-Degree of innovation	
	-Collaboration and leadership	
	-Implementation of patients	
	-Pressure of different interest groups	
	-Environmental impact	

Main references used: EVIDEM tool [10, 11] and Guindo [13], Golan [14], Tanios [15], Tromp [16] studies

The current work includes eight studies that address the identification, selection or classification of criteria used in the international arena for the establishment of health priorities. [10–16] Three of the studies retrieved refer to the EVIDEM (Evidence and Value: Impact on Decision Making) tool. In this multicriteria tool, developed from a thorough analysis of the literature, the opinion of experts and different international experiences, refers to 13 universal quantitative criteria (5 domains) and 7 contextual qualitative criteria (3 domains). [10–12]

## Discussion

The results of our review show that, despite a general agreement regarding the need to establish rational and transparent procedures to prioritize medicinal products and medical devices, and a certain concordance with respect to critical domains exists, there is scarce information available on the explicit processes employed by the evaluation agencies for the establishment of priority issues [26–29].

This review can be used by different bodies interested in priorization framework. All domains and criteria have advantages and limitations, despite the fact all themes were devised rigorously. It should be noted that the present review is limited by the difficulties inherent in the bibliographic search. To improve efficiency, the search has been restricted to the title, so it is possible to have lost some relevant article despite using different combinations of keywords and related terms. In addition, in many cases this type of information is not published in scientific journals and is difficult to recover due to the serious shortcomings of the search engines of web pages, or because it is published in other languages. In any case, we believe that this would not detract from the current work, since we do not intend to describe all international experiences, but to identify those criteria and elements that may be key to the development of a national prioritization proposal. The implementation science principles for pharmacist and other healthcare providers, discovering and applying strategies designed to incorporate evidence-based interventions into routine practice is a must [30].

## Conclusion

Our review provides a thorough analysis of the relevant issues and offers key recommendations regarding considerations for developing a national prioritization framework. Findings are envisioned to be useful for different public organizations that are aiming to establish healthcare priorities.

## Additional file

Additional file 1: Search strategy. (DOCX 11 kb)
