# Supplementary material for: Systematic review for the development of a pharmaceutical and medical products prioritization framework
Source: J Pharm Policy Pract. 2019 Aug 21;12:21. doi: 10.1186/s40545-019-0181-2 (PMC6702737; doi:10.1186/s40545-019-0181-2)
Supplement: Supplementary file 1 — Search strategy. (DOCX 11 kb) [file 40545_2019_181_MOESM1_ESM.docx]

Appendix: Search strategy

A. Manual search in International Journal of Technology Assessment in Health Care (Cambridge Journals) 22 references

Strategy line:

Items 1: priority or prioritization or prioritization or prioritize or prioritise or priorities

B. Inahta

Strategy line:

Item 1: priority or prioritization or prioritisation or prioritize or prioritise or priorities

Item 2: criteria or setting or settings or set or approach or approaches or procedure or procedures

Item 3: #1 OR #3 2

C. General databases: PubMed (Medline) 229 references.

Strategy line:

Item 1: “Biomedical Technology”[Mesh] OR “Technology Assessment, Biomedical”[Mesh] OR “Technology”[Mesh] OR technologies[TI] OR technology[TI] OR intervention*[TI] OR HTA[TI]

Item 2: priority[Title] OR prioritization[Title] OR prioritisation[Title] OR prioritize[Title] OR prioritise[Title] OR priorities[Title] OR selection [Title]

Item 3: criteria[Title] OR setting[Title] OR settings[Title] OR set[Title] OR approach[Title] OR approaches[Title] OR procedure[Title] OR procedures[Title]

Item 4: #1 AND #2 AND #3

Embase 401 references.

Strategy line:

Item 1: (medical technology or biomedical technology assessment or technology) ti

Item 2: (technologies or technology or intervention* or hta) ti.

Item 3: #1 OR #2

Item 4: (priority or prioritization or prioritisation or prioritize or prioritise or priorities) ti.

Item 5: (criteria or setting or settings or set or approach or approaches or procedure or procedures) ti.

Item 6: #3 AND #4 AND #5
